# Silicon and Plants: Current Knowledge and Technological Perspectives

**DOI:** 10.3389/fpls.2017.00411

**Published:** 2017-03-23

**Authors:** Marie Luyckx, Jean-Francois Hausman, Stanley Lutts, Gea Guerriero

**Affiliations:** ^1^Groupe de Recherche en Physiologie Végétale, Earth and Life Institute - Agronomy, Université Catholique de LouvainLouvain-la-Neuve, Belgium; ^2^Environmental Research and Innovation Department, Luxembourg Institute of Science and TechnologyEsch-sur-Alzette, Luxembourg

**Keywords:** silicic acid, biosilicification, stresses, priming, cell wall

## Abstract

Elemental silicon (Si), after oxygen, is the second most abundant element in the earth’s crust, which is mainly composed of silicates. Si is not considered essential for plant growth and development, however, increasing evidence in the literature shows that this metalloid is beneficial to plants, especially under stress conditions. Indeed Si alleviates the toxic effects caused by abiotic stresses, e.g., salt stress, drought, heavy metals, to name a few. Biogenic silica is also a deterrent against herbivores. Additionally, Si ameliorates the vigor of plants and improves their resistance to exogenous stresses. The protective role of Si was initially attributed to a physical barrier fortifying the cell wall (e.g., against fungal hyphae penetration), however, several studies have shown that the action of this element on plants is far more complex, as it involves a cross-talk with the cell interior and an effect on plant metabolism. In this study the beneficial role of Si on plants will be discussed, by reviewing the available data in the literature. Emphasis will be given to the protective role of Si during (a)biotic stresses and in this context both priming and the effects of Si on endogenous phytohormones will be discussed. A whole section will be devoted to the use of silica (SiO_2_) nanoparticles, in the light of the interest that nanotechnology has for agriculture. The paper also discusses the potential technological aspects linked to the use of Si in agriculture and to modify/improve the physical parameters of plant fibers. The study indeed provides perspectives on the use of Si to increase the yield of fiber crops and to improve the thermal stability and tensile strength of natural fibers.

## Introduction

Silicon (Si) is considered non-essential (or quasi-essential, Epstein and Bloom, 2005) for plant growth and development. Plants develop well in its absence, although in some cases, e.g., the silicifier horsetail and rice, the absence of Si triggers increased susceptibility to fungal infection (Datnoff and Rodrigues, 2005; Law and Exley, 2011). When supplied to the growth medium (as silicic acid, *vide infra*), plant vigor and resistance to (a)biotic stresses increase (Azeem et al., 2015; Coskun et al., 2016; Guerriero et al., 2016a). Si is taken up by plants as silicic acid Si(OH)_4_ via aquaporin type channels (Nod26-like intrinsic proteins, NIPs) (Ma et al., 2006; Grégoire et al., 2012; Deshmukh et al., 2013). A specific 108 amino acid spacing between the conserved NPA domains determines Si(OH)_4_ permeability (Deshmukh et al., 2015). Plants are classified into accumulators, excluders and intermediate type (Mitani and Ma, 2005), depending on the amount of biogenic silica found in their tissues. Among the accumulators are Equisetales, Cyperales and Poales: in *Graminae*, rice is the highest silicifier where Si (in the form of biogenic silica, *vide infra*) accounts for up to 10% of the shoot dry weight (Ma et al., 2002). Tomato is among the excluders, while *Urtica dioica* (i.e., nettle) is an intermediate type (Trembath-Reichert et al., 2015).

In (some) plants the provision of Si(OH)_4_ has a latent effect in the absence of an external stimulus (Fauteux et al., 2005, 2006). This has been observed in the *Arabidopsis*-powdery mildew pathosystem (Fauteux et al., 2006). It should, however, be noted that in rice, Si(OH)_4_ supplementation does trigger major changes, as it induces the upregulation and downregulation of 35 and 121 transcription factors respectively (Van Bockhaven et al., 2012). This difference may be in part due to the different cell wall types (Yokoyama and Nishitani, 2004) and to the structural importance of Si in type II cell walls (i.e., cell walls characterized by the presence of more phenylpropanoids as compared to type I cell walls in dicots).

By precipitating as SiO_2_ and being incorporated into biological structures (e.g., the cell wall, *vide infra*), Si exerts its protective action via the formation of a physical barrier. However, this passive role is too simplistic and does not explain why plants supplemented with Si are better suited to face exogenous stresses. Compelling evidence in the literature shows that specific cell wall components trigger SiO_2_ precipitation (reviewed by Guerriero et al., 2016a). In rice cell suspension culture, a hemicellulose-bound form of Si has been identified (He et al., 2015), in horsetail mixed-linkage glucans (MLGs) have been proposed to participate in SiO_2_ formation (Fry et al., 2008) and this has been recently confirmed in rice where overexpression of a hydrolase acting on MLGs was shown to affect silicification (Kido et al., 2015). In horsetail, callose was shown to template biosilicification (Law and Exley, 2011). Very recently, the role of callose in templating biosilicification has been additionally proven by using *Arabidopsis* plants either overexpressing or lacking the callose synthase gene *PMR4* (Brugiére and Exley, 2017): while the wild-type plants and overexpressors responded to a pathogen-like challenge by accumulating both callose and silica, the mutants did not produce callose and, consequently, deposited significantly less silica.

## Si Priming

Several papers demonstrated that Si(OH)_4_ (hereafter referred to as Si for simplicity) acts as a “tonic” by priming plants, i.e., by preparing the defense responses which are then fully deployed at the onset of the stress, as will be discussed in detail in the next sections. The effects of Si under normal conditions are indeed latent, since, for the majority of the studies available, no major modifications, e.g., in gene expression, are observed. Under control conditions Si probably activates the metabolic status of the plant, by making it more efficient in responding to exogenous stimuli.

In rice, a Si-accumulator, Si causes alterations of C/N balance in the source-sink relationship under unstressed conditions, by favoring a remobilization of amino acids to support the increased N demand during grain development (Detmann et al., 2012, 2013). These data support the hypothesis that Si has a signaling role in plant cells. Si was indeed suggested to have a role as second messenger by binding to the hydroxyl groups of proteins involved in cell signaling, thereby partaking in the signal transduction (Fauteux et al., 2005).

It is important to mention that Si primes defense responses also in Si non-accumulators, i.e., tomato (Ghareeb et al., 2011): tomato is protected against *Ralstonia solanacearum* by Si which causes an upregulation, upon infection, of genes involved in ethylene and jasmonic acid signaling, i.e., *JERF3*, *TRSF1*, *ACCO*, as well as genes involved in stress response, i.e., trehalose phosphatase, late embryogenesis abundant protein, ferritin. In this study, the authors also observed an increased expression of a negative regulator of the jasmonic acid signal, *JAZ1*, together with a ubiquitin protein-ligase: the authors propose that *JAZ1* helps in preventing the eventual damage caused by the stimulation of defense-related compounds and that the ubiquitin protein-ligase may degrade JAZ1. In tomato challenged by *R. solanacearum*, Si also upregulates a MAPK (*MAPK19*), a WRKY transcription factor and linker histones (*H1* and *H5*). These findings corroborate the role of Si in intracellular signaling and suggest its involvement in transcription too (Ghareeb et al., 2011).

Silicon was shown to upregulate the expression of a leucine-rich repeat receptor-like kinase (LRR-RLK) in rice (Fleck et al., 2011), which is a protein involved in intracellular signal transduction. High-throughput technologies relying on *–omics* will help shed light on the missing genes/proteins involved in the signal transduction underlying Si priming (the so-called “prime-omics”; Balmer et al., 2015).

## Si and Abiotic Stress Alleviation

Si assumes key functions in the plant response to numerous environmental constraints. Two major processes contributing to stress resistance are commonly considered (i) a physical and mechanical protection afforded by SiO_2_ deposits and (ii) a biochemical response triggering metabolic changes. The precise distribution/speciation of accumulated Si in plant tissue allows us to gain additional information regarding its modalities of action and requires the use of biophysical tools, such as laser ablation (LA), extended X-ray fine structure (EXAFS), X-ray absorption near edge structure (XANES) and micro particle-induced X-ray emission (micro-PIXE).

According to Liang et al. (2013), Si improves lodging resistance by strengthening the stem basis in rice. It also enhances UV tolerance due to the protective effect of Si deposition bodies on the leaf epidermis (Goto et al., 2003) or by reducing UVB-induced membrane damages (Shen et al., 2010).

Silicon influences water relations in drought-treated plants: it induces the formation of a silica cuticle double layer under the leaf epidermis which reduces water losses through cuticular transpiration (Gong et al., 2003). Si also reduces stomatal conductance in relation to turgor loss of guard cells resulting from Si deposition and modified cell wall properties (Zhu and Gong, 2014). Si improvement of drought resistance may also be ascribed to strong abilities to extract water from the soil as a consequence of Si-related promotion of root elongation (Hattori et al., 2005) and up-regulation of aquaporin genes (Liu et al., 2015).

Silicon contributes to salt stress alleviation through inhibition of Na^+^ (Zhu and Gong, 2014) and Cl^-^ (Shi et al., 2013) uptake. Translocation of toxic ions from root to shoot is also reduced by Si supply (Savvas and Ntatsi, 2015). In rice, Si alleviates NaCl toxicity by blocking the transpirational bypass flow through precipitation as SiO_2_ in exodermis and endodermis (Yeo et al., 1999). Potassium uptake allowing the maintenance of K/Na is improved by Si nutrition which has a direct stabilizing effect on proton pump activity in salt-treated root tips (Xu et al., 2015).

In metal-polluted soil, Si may influence the bioavailability of toxic elements. The presence of soil sodium metasilicate or alkaline Si-containing material may induce a rise in the rhizospheric pH leading to a decrease in available heavy metal concentration in the soil (Wu et al., 2013). Soluble silicate hydrolyzes to generate gelatinous metasilicic acid (H_2_SiO_3_) retaining heavy metals (Gu et al., 2011). According to Kidd et al. (2001), Si-treated plants may also exude phenolics such as catechin and quercetin having strong Al-chelating abilities. The formation of hydroxyl-aluminum silicate in the apoplast also contributes to Al detoxification (Wang et al., 2004).

Compartmentation of toxic ions is an important process in heavy metal tolerance. Si improves heavy metal retention by roots, with an obvious accumulation in the endodermis (Keller et al., 2015). At the shoot level, accumulation of Mn was mainly observed in epidermis in response to Si treatment (Doncheva et al., 2009). Iwasaki and Matsumura (1999) reported that Si increases Mn accumulation in the leaf trichomes. Controversial data are available in the literature regarding co-precipitation of Si with heavy metals. Keller et al. (2015) did not detect Cu and Cd in phytoliths and the absence of Cu-Si coprecipitation was also noticed in maize by Collin et al. (2014). He et al. (2013), however, identified a mechanisms of co-deposition of Si and Cd in the rice cell walls via a [Si-wall matrix] Cd complexation, which may explain a Si-induced decrease in the Cd influx in cells. Ma et al. (2015) considered that a hemicellulose bound form of Si with a net negative charge is responsible for inhibition of Cd uptake leading to a downregulation of *Nramp5* coding for a transporter involved in Cd transport. Kim et al. (2014) also reported a downregulation of other heavy metals transporter (*OsHMA2* and *OsHMA3*) when Cu/Cd-treated rice was supplied by Si.

Numerous studies reported that Si induces an improved behavior of heavy metal-treated plants in relation to regulation of antioxidant enzymes (Adrees et al., 2015), oversynthesis of endogenous antioxidants leading to mitigation of oxidative stress (Imtiaz et al., 2016), maintenance of net photosynthesis relying on the stabilization of chloroplast structures, PSII integrity and increased pigment concentration (Nwugo and Huerta, 2008; Tripathi et al., 2015a). Si may thus be of paramount importance for triggering adapted plant response, but the precise molecular cues involved in the adaptative processes still need to be clearly identified.

## Si and Biotic Stress

Si was reported to improve defense against biotic constraints occurring in the form of plant pathogens (fungi, bacteria, and viruses) or animals (vertebrates and arthropod herbivores).

Silicon deposition increases abrasiveness of plant tissues and thus reduces palatability and digestibility for herbivores (Massey and Hartley, 2009). Hartley et al. (2015) demonstrated by Scanning electron microscopy with energy dispersive X-ray spectroscopy (SEM-EDX) that phytolith morphology inside the tissues has more influence on abrasiveness than the actual Si concentration. Using the same technique, Keeping et al. (2009) demonstrated that the pattern of Si deposition in sugarcane is responsible for enhanced resistance to *Eldana saccharina*. Physical strength of the leaf resulting from Si accumulation may afford mechanical protection and thus lower the rate of infection as reported for *Rhizoctonia solani* (Zhang et al., 2013; Schurt et al., 2014) or *Bipolaris oryzae* (Ning et al., 2014).

Biochemical/molecular mechanisms are also induced or re-inforced by Si allowing the plant to improve resistance to biotic stress and include defensive compounds such as phenolics, phytoalexins and momilactones (Remus-Borel et al., 2005), but also to activation of defensive enzymes such as peroxidase, polyphenol oxidase, lipoxygenase and phenylalanine ammonia lyase (Rahman et al., 2015). According to Cai et al. (2008), Si treatments may increase transcripts levels corresponding to those defense-related genes.

Reynolds et al. (2016) reported that Si also operates by attracting predators or parasitoids to plant under herbivore attack. Indeed, soluble Si contributes to increase herbivore-induced plant volatiles to promote predator attraction by pest-infected plants. Moreover, according to James (2003) and Connick (2011), the phenology of insect’s life cycle is also slowed down in Si-treated plants, making it more prone to predation.

## Effects of Si on Phytohormones

Silicon impacts on endogenous phytohormones are commonly analyzed in response to stress conditions. In rice plants exposed to heavy metals, Si reduced endogenous concentration of jamonic acid (JA) and salicylic acid (SA), while abscisic acid (ABA) first increased and then decreased after 14 days of treatment (Kim et al., 2014): the ABA has an antagonist behavior with JA/SA biosynthesis. The effect of such phytohormonal changes on the expression of genes involved in heavy metal response still needs to be elucidated in Si-treated plants. Kim et al. (2011) also reported that Si reduced JA concentration in response to wounding, while Lee et al. (2010) reported an increase in gibberellins concentration in Si-treated plants exposed to salinity.

Resistance to biotrophic pathogens may be associated with SA whereas JA and ethylene (ET) are generally associated with resistance to necrotrophic pathogens. Fauteux et al. (2006) showed that Si improved biosynthesis of SA, JA and ET in leaves exposed to *Erysiphe cichoracearum*. Similarly, Si-treated tomato plants exposed to *R. solanacearum* activated JA and ET signaling pathways to increase resistance (Ghareeb et al., 2011). Brunings et al. (2009) also provided evidence that genes controlling ET signaling pathway may be activated by Si treatment. Conversely, Si improves resistance to the fungus *Cochliobolus miyabeanus* by interfering with the production of fungal ET (Van Bockhaven et al., 2015). Data regarding the effect of Si on phytohormone metabolism in the absence of stress are still rare. Markovich et al. (2017), however, recently demonstrated that Si increases cytokinin biosynthesis in *Sorghum* and *Arabidopsis* and that such an increase may strongly contribute to delay senescence. Plant hormones interactions are responsible for a complex biochemical and physiological network: a deep understanding of Si influence on hormonal properties thus requires technical approaches allowing to quantify a wide range of hormonal compounds simultaneously, including minor conjugated forms.

## Silica Nanoparticles

The use of nanotechnology in agriculture is gaining importance because it contributes to develop new sustainable strategies. Nanomaterials can for example be engineered to immobilize nutrients or to release them in a controlled manner in the soil (Fraceto et al., 2016).

Some papers in the literature have studied the effects of silica nanoparticles (SNPs) on plant physiology and we will here review some of them.

Mesoporous SNPs (MSNPs, 20 nm in size) coupled to FITC were shown to be taken up by three important crops (lupin, wheat, maize), as well as *Arabidopsis* protoplasts and to be translocated to the aerial parts following the xylematic flow after entering the roots via symplastic/apoplastic routes (Sun et al., 2014). Very interestingly, this study also showed that MSNPs accumulated in the cell walls, therefore highlighting the existence of an affinity with cell wall components. The monodisperse nature of the MSNPs and their size, achieved via a fine-tuning of pH and surfactant concentration, were essential for the efficient uptake by plants: the entry takes place via the pores in the cell walls of the roots cells (Sun et al., 2014).

Mesoporous SNPs were shown to boost the growth, total protein content and photosynthesis of lupin and wheat seedlings and to induce no changes in the activity of antioxidant enzymes (Sun et al., 2016). Interestingly in this study, the authors observed a shift of 14 cm^-1^ and 10 cm^-1^ in the Raman peaks of chlorophyll from wheat and lupin when isolated chloroplasts were incubated with MSNPs suggesting a change in the molecular structure of chlorophyll.

Silica nanoparticles were shown to protect wheat seedlings against UV-B stress by stimulating the antioxidant defense system (Tripathi et al., 2016). In particular, SNPs reduced the adverse effects of the UV-B stress, i.e., low fresh weight, reduction in chlorophyll and tissue damage. Since the levels of nitric oxide reached a peak after UV-B+SNPs treatment, a protective role via the modulation of NO levels was proposed by the authors.

Silica nanoparticles also conferred protection via mitigation of oxidative stress in pea seedlings treated with Cr(VI): the activities of enzymes such as superoxide dismutase, ascorbate peroxidase increased significantly in the presence of SNPs, while catalase, glutathione reductase and dehydroascorbate reductase were less inhibited by Cr(VI) in the presence of SNPs (Tripathi et al., 2015b).

Silica nanoparticles (12 nm) were also found to improve germination in a known Si-excluder, tomato: at a concentration of 8 g/L, SNPs improved seedling germination, as well as fresh and dry weight by 116.6 and 117.5% respectively (Siddiqui and Al-Whaibi, 2014).

Nanostructured SiO_2_ (TMS) was shown to be valuable in larch seedling production, because, when applied to the roots of 1-year-old seedlings via soaking for 6 h, it promoted lateral root growth, main root length and chlorophyll content (Bao-shan et al., 2004).

The effect of SNPs was, however, shown to be dependent on the plant species, as in Bt-transgenic cotton they significantly decreased plant growth (Le et al., 2014). SNPs toxicity may be linked to pH and nutrient adsorption problems. Indeed, in thale cress, SNPs phytotoxicity was triggered when the pH of the medium was not adjusted or silanol groups were not removed from the surface (Slomberg and Schoenfisch, 2012). The alkaline pH (pH 8 ca.) makes nutrients less available for uptake, while the negatively charged SNPs tend to adsorb nutrients.

## Si and Fiber Crops

Fiber crops like textile hemp (*Cannabis sativa* L.) are natural resources which provide long and strong cellulosic fibers (a.k.a. bast fibers) used in both the textile and biocomposite sectors (Guerriero et al., 2014; Andre et al., 2016; Guerriero et al., 2016b). Given the positive effects of Si on plants, its use for fiber crop growth may provide an enhanced biomass yield and, therefore, an increased production of bast fibers. The association of SiO_2_ with the fiber cell walls may provide new properties, notably and increased durability. In this respect, it should be noted that hemp woody fibers, which contain SiO_2_ and therefore bind well with lime, are already used to manufacture a lightweight concrete-like material used in eco-construction and known as hempcrete. The few studies available on the specific Si impact on fiber crops confirm protection against abiotic stresses. In ramie [*Boehmeria nivea* (L.) Gaud.], the application of Si ameliorated Cd toxicity via stimulating the activities of antioxidant enzymes (Tang et al., 2015). Bakry et al. (2015) and Shedeed et al. (2016) reported that foliar application of Si improved the nutrient status of flax and increased straw and oil yield/plant.

Silicon accumulation in fiber crops is genetically controlled, as demonstrated for bamboos by Collin et al. (2012). Exogenous Si did not reduce Cu absorption by bamboos growing on contaminated solution, but reduced toxicity symptoms (Collin et al., 2013, 2014). Si also improved the growth of cotton exposed to Cd but, in this case, Si reduced Cd uptake and mitigated the adverse effect of this heavy metal by improving plant growth, biomass and photosynthetic parameters in stressed plants (Farooq et al., 2013).

Data concerning the direct influence of Si on fiber development itself are crucially lacking. Some old studies, however, provided indirect evidences that Si may assume important functions in this respect. Khan and Roy (1964) reported that soil application of silicate improved the size of the commercial fiber jute by increasing cell elongation and fineness. According to Boylston (1988), the Si concentration is high during the elongation phase of cotton fiber development but decreases as the fiber matures. The ratio of the amount of Si per mass of fiber peaks at the time when secondary wall initiation occurs (Boylston et al., 1990). Si is known to interact with cell walls (see Introduction), although the mechanisms underlying the final incorporation of polymerized Si into the cell wall remain elusive. Kido et al. (2015) recently demonstrated that the interaction of mixed linkage glucan (1;3, 1;4)-β-D-glucan with Si may have obvious mechanical consequences.

**FIGURE 1:**
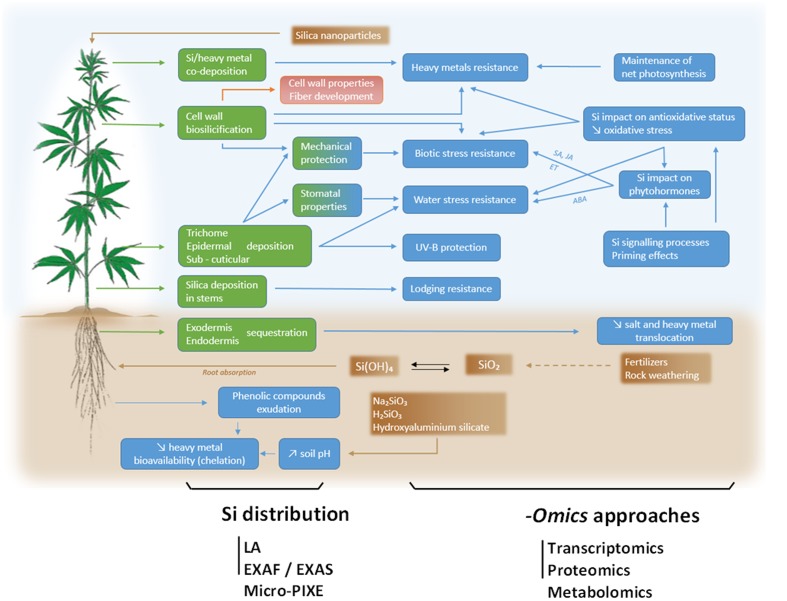
**Global overview of Si impact on hemp (*Cannabis sativa* L.), here depicted as a model plant in light of its economic importance as a source of bast fibers**. Speciation of Si in soil and application of SiO_2_ nanoparticles are indicated in brown boxes and possible sites of Si deposit in the plant are indicated in green boxes. Resulting consequences of Si accumulation in terms of stress resistance and underlying physiological processes are indicated in blue boxes. For further details, please refer to the text. A deep understanding of processes involved in Si absorption, translocation and physiological consequences require holistic *-omics* approaches including transcriptomics, proteomics and metabolomics tools. The precise Si distribution may be assessed by laser ablation (LA), extended X-ray fine structure (EXAFS), X-ray absorption near edge structure (XANES) and micro particle-induced X ray emission (micro-PIXE).

Si beneficial influence on natural fiber properties is confirmed by the use of Si-containing compounds during industrial processing of harvested fibers. Natural fibers are gaining attention in engineering composite industry. However, cell wall polymers often bear hydrophilic hydroxyl groups able to form new hydrogen bonds with water molecules, which hinder hydroxyl group to react with the polar matrix of the composites (Mwaikambo and Ansell, 2002). Silane is an inorganic compound (SiH_4_) commonly used to improve tensile strength and thermal stability of natural fibers (Abdelmouleh et al., 2004) which may be due to the emergence of Si-O-C and Si-O-Si links on the cellulose surface (Lu et al., 2013). Other Si treatment, including siloxane and nano Si dioxide are also used for similar purposes (Kabir et al., 2012; Siengchin and Dangtungee, 2014; Orue et al., 2016). It may thus be hypothesized that Si treatment *in vivo* during fiber development (and not only *in vitro* on harvested mature fibers) may lead to several promising application. This exciting goal, however, requires a multidisciplinary approach to gain a better understanding of Si influence on the modalities of fiber development (**Figure 1**).

## Conclusion and Future Perspectives

Silicon is an abundant element on Earth and its positive effects on plants make it important in agriculture. The study of the Si-plant binomium has still much to teach us and this is particularly the case for e.g., the cell wall-related mechanisms underlying its prophylactic role under stress. The plant cell wall takes active part in the response to (a)biotic stresses by establishing a signaling cascade toward the cell interior (Hamann, 2015) and by undergoing a remodeling (Tenhaken, 2014). It is therefore clear that part of the beneficial effects of Si on plants is linked to direct/indirect effects on the cell wall.

In the future, research activities centered on specific aspects of the interaction Si-plants will be important to devise agricultural strategies aimed at improving crop yield.

## Author Contributions

GG conceived the idea of writing the paper. ML, J-FH, SL, and GG collected the literature data and wrote the manuscript.

## Conflict of Interest Statement

The authors declare that the research was conducted in the absence of any commercial or financial relationships that could be construed as a potential conflict of interest.
